# Structure of SARS-CoV-2 MTase nsp14 with the inhibitor STM957 reveals inhibition mechanism that is shared with a poxviral MTase VP39

**DOI:** 10.1016/j.yjsbx.2024.100109

**Published:** 2024-07-29

**Authors:** Eva Zilecka, Martin Klima, Milan Stefek, Milan Dejmek, Radim Nencka, Evzen Boura

**Affiliations:** Institute of Organic Chemistry and Biochemistry, Academy of Sciences of the Czech Republic, v.v.i, Flemingovo nám. 2, 166 10 Prague 6, Czech Republic

**Keywords:** Methyltransferase, Inhibitor, Crystal structure

## Abstract

Nsp14 is an RNA methyltransferase (MTase) encoded by all coronaviruses. In fact, many viral families, including DNA viruses, encode MTases that catalyze the methylation of the RNA precap structure, resulting in fully capped viral RNA. This capping is crucial for efficient viral RNA translation, stability, and immune evasion. Our previous research identified nsp14 inhibitors based on the chemical scaffold of its methyl donor − the S-adenosyl methionine (SAM) − featuring a modified adenine base and a substituted arylsulfonamide. However, the binding mode of these inhibitors was based only on docking experiments. To uncover atomic details of nsp14 inhibition we solved the crystal structure of nsp14 bound to STM957. The structure revealed the atomic details of nsp14 inhibition such that the 7-deaza-adenine moiety of STM957 forms specific interactions with Tyr368, Ala353, and Phe367, while the arylsulfonamide moiety engages with Asn388 and Phe506. The large aromatic substituent at the 7-deaza position displaces a network of water molecules near the adenine base. Surprisingly, this was recently observed in the case of an unrelated monkeypox MTase VP39, where the 7-deaza modified SAH analogs also displaced water molecules from the vicinity of the active site.

## Highlights

• A crystal structure of coronaviral methyltransferase (MTase) nsp14 was solved with an inhibitor STM957.
• The structure revealed that STM957 is, indeed, a bisubstrate inhibitor.
• The aromatic moiety at the 7-deaza base of STM957 replaced a water network near the adenine base.
• Similar mechanism of inhibition was observed in mpox MTase VP39.
• Displacement of water molecules from the vicinity of the active site might be a common mechanism of viral MTase inhibitors.

## Graphical abstract

**Figure:**
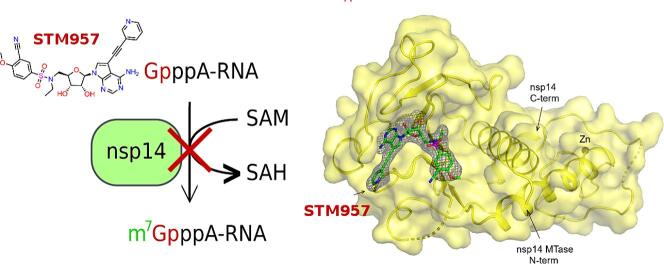

## Introduction

Many distant viral families such as poxviruses or coronaviruses encode RNA methyltransferases (MTases) that catalyze methylation of the precap structure on the 5′ end of RNA leading to a fully capped viral RNA (Nencka et al., 2022, Shuman, 2015, Skvara et al., 2023). This process was discovered, in part, thanks to poxviruses and it was established that RNA capping is essential for efficient translation of viral RNA (Meade et al., 2019, Shuman and Hurwitz, 1981). Later, it was also established that RNA capping is important for the stability of RNA and for immune evasion (Beachboard and Horner, 2016). In fact, human innate system has many receptors for patterns of viral origin including viral RNA or DNA. For instance, RIG-I and MDA5 detect 5′ unphosphorylated or phosphorylated RNA and their activation leads to the expression of interferon stimulated genes (Brisse and Ly, 2019). These include the IFIT proteins that recognize RNA molecules that are not properly capped and prevent their translation (Fensterl and Sen, 2015). Therefore, many viruses evolved a capping machinery that includes one or two MTases. In coronaviruses, that is the nsp16 MTase that methylates the 2′-O-ribose position (Decroly et al., 2011, Dostalik et al., 2021) of the first nucleotide and nsp14 MTase that methylates the N7 position of the guanosine of the precap structure (Bouvet et al., 2010).

**Fig. 1:**
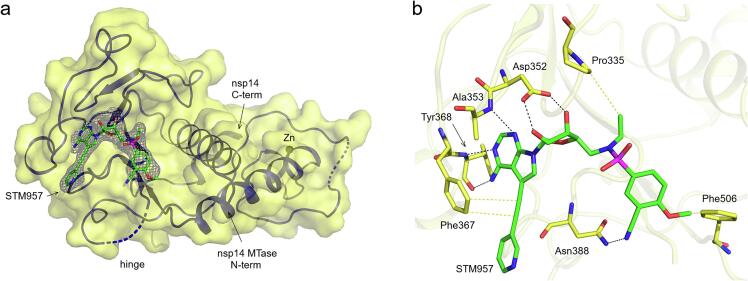
Crystal structure of SARS-CoV-2 nsp14 MTase domain in complex with the STM957 inhibitor. a, Overall view of the nsp14/STM957 complex. The protein backbone of the nsp14 MTase domain is shown in cartoon/surface representation. The STM957 ligand is shown in stick representation and colored according to elements: carbon, green; nitrogen, blue; oxygen, red; sulphur, magenta. The Fo-Fc omit map contoured at 2σ is shown around the STM957 ligand. The TELSAM crystallization tag N-terminally fused to the nsp14 MTase domain is not shown. b, Detailed view of the STM957 ligand binding site. The STM957 ligand and selected nsp14 amino acid residues are shown in stick representation with carbon atoms colored according to the protein assignment and other elements colored as in (a). Selected hydrogen bonds and hydrophobic interactions involved in the nsp14 -STM957 interaction are depicted as dashed black and yellow lines, respectively. Hydrogen atoms are not shown. (For interpretation of the references to colour in this figure legend, the reader is referred to the web version of this article.)

Coronaviral nsp14, similarly to most MTases, uses S-adenosyl methionine (SAM) as the donor of the methyl group. We have previously prepared a series of nsp14 inhibitors (Stefek et al., 2023) that chemically resemble SAM except that the adenine base is modified and has a large substituent at the 7-deaza position and the amino acid moiety is replaced by a substituted arylsulfonamide such as the compound STM957 (Fig. 1A). However, how exactly these compounds inhibit nps14 was not known although docking experiments suggested the large aromatic substituent occupies a large cavity close to the binding site for the adenine base of the SAH molecule (Otava et al., 2021) and the arylsulfonamide moiety occupies the RNA binding site (Ahmed-Belkacem et al., 2022, Jung et al., 2022). In this study, we describe the precise mechanism in atomic detail of nps14 inhibition by this class of SAH analogs.

## Materials and methods

### Protein expression and purification

The SARS-CoV-2 nsp14 MTase domain was expressed and purified as described previously (Nigam et al., 2024). Briefly, the nsp14 MTase domain (GenBank: YP_009725309.1, residues 300–527) was expressed as a fusion protein with an *N*-terminal hexahistidine (His_6_) purification tag followed with a SUMO solubility and folding tag, and a TELSAM crystallization tag (Kottur et al., 2022). The fusion protein was expressed in the *E. coli* BL21 DE3 NiCo bacterial strain (New England Biolabs) in the autoinduction ZY-5052 medium. The cells were harvested by centrifugation, resuspended in the lysis buffer (50 mM Tris.HCl pH 8.0, 400 mM NaCl, 20 mM imidazole, 10 mM MgCl_2_, 10 µM ZnCl_2_, 3 mM β-mercaptoethanol, and 250U of DNA endonuclease DENERASE (c-LEcta)), and sonicated using the Q700 Sonicator instrument (QSonica). The lysate was precleared with centrifugation for 30 min at 30,000 g and incubated with the HisPur Ni-NTA Superflow agarose (Thermo Fisher Scientific) for 60 min. Then, the agarose beads were extensively washed with the lysis buffer and the protein was eluted with the lysis buffer supplemented with 300  mM imidazole. The eluted fusion protein was then treated with Ulp1 protease to cleave the His_6_-SUMO tag. The TELSAM-nsp14 MTase protein was further purified using the size exclusion chromatography at the HiLoad 16/600 Superdex 200 prep grade column (Cytiva) pre-equilibrated with the size-exclusion buffer (25 mM Tris.HCl pH 8.3, 200 mM KCl, and 2 mM TCEP). Fractions containing the purified TELSAM-nsp14 MTase protein were concentrated to 4 mg/ml, flash frozen in liquid nitrogen, and stored at 193 K.

### Crystallization and crystallographic analysis

For the crystallographic analysis, the TELSAM-nsp14 MTase protein was supplemented with two fold molar excess of the STM957 ligand. Protein crystals were obtained within 2 days at 18 °C in a sitting drop consisting of 200 nl of the protein and 200 nl of the mother liquor using the vapor diffusion method at 291 K. The well solution was composed of 12.5 % w/v PEG 4000, 20 % w/v 1,2,6-hexanetriol, 100 mM Gly-Gly/AMPD pH 8.5, 10 mM spermine, 10 mM spermidine, 10 mM 1,4-diaminobutane, and 10 mM DL-ornithine. The crystals were harvested and cooled in liquid nitrogen. The crystallographic dataset was collected from a single crystal on the BL14.1 beamline at the BESSY II electron storage ring operated by the Helmholtz-Zentrum Berlin (Mueller et al., 2015).

The data were integrated and scaled using XDSapp v3.1.9 (Kabsch, 2010, Sparta et al., 2016). The structure of the TELSAM-nsp14 MTase/STM957 complex was solved by molecular replacement using the structure of the TELSAM-nsp14 MTase/SS148 complex as a search model (pdb entry 8BWU) (Nigam et al., 2024). The initial model was obtained with Phaser v2.8.3 (McCoy et al., 2007). The model was further improved using automatic model refinement with the phenix.refine tool (Afonine et al., 2012) from the Phenix package v1.20.1-4487 (Liebschner et al., 2019) and manual model building with Coot v0.9.8.7 (Debreczeni and Emsley, 2012). Geometrical restraints for the ligand were generated with Grade2 v1.3.1 (Global Phasing Ltd.). Statistics for data collection and processing, structure solution and refinement are summarized in Table 1. Structural figures were generated with the PyMOL Molecular Graphics System v2.5.4 (Schrödinger, LLC). The atomic coordinates and structural factors were deposited in the Protein Data Bank (https://www.rcsb.org) under the accession code 9FEH.

**Table 1:** Statistics for data collection and processing, structure solution and refinement of the crystal structure of the TELSAM/SARS-CoV-2 nsp14 MTase/STM957 complex. Numbers in parentheses refer to the highest resolution shell. R.m.s.d., root-mean-square deviation.

**Crystal**		**nsp14 + STM957**
PDB accession code		9FEH
**Data collection and processing**		
Space group		P 65
Cell dimensions	a, b, c (Å)	109.4 109.4 48.8
	α, β, γ (°)	90.0 90.0 120.0
Resolution range (Å)		47.36–1.99 (2.06–1.99)
No. of unique reflections		23,077 (2,291)
Completeness (%)		99.9 (99.9)
Multiplicity		20.5 (21.0)
Mean I/σ(I)		7.42 (0.48)
Wilson B factor (Å^2^)		37.13
R-merge		0.3426 (3.814)
R-meas		0.3512 (3.908)
CC1/2 (%)		99.7 (44.9)
CC* (%)		99.9 (78.8)
**Structure solution and refinement**		
R-work (%)		21.58 (38.66)
R-free (%)		23.03 (37.84)
CC-work (%)		95.9 (66.7)
CC-free (%)		97.1 (59.3)
R.m.s.d.	bonds (Å)	0.004
	angles (°)	0.62
Average B factor (Å^2^)	overall	46.12
	protein	46.35
	ligands	39.02
	solvent	44.66
Clashscore		1.18
Ramachandran (%)	favored	98.1
	allowed	1.9
	outliers	0.0

## Results and discussion

To uncover atomic details of the nsp14/STM957 interaction, we carried out a crystallographic analysis of the nsp14/STM957 complex. We used the nsp14 MTase domain fused to a small crystallization tag TELSAM (Kottur et al., 2022). The obtained crystals belonged to the hexagonal P6_5_ space group and diffracted to 2.0 Å resolution. The structure was subsequently solved by molecular replacement (detailed in SI) and further refined to good R factors and geometry as summarized in Table 1**.**

The electron density for the STM957 ligand was well-defined and the ligand was bound to the SAM-binding site of nsp14 as expected (Fig. 1). The nsp14-STM957 interaction is mediated by multiple hydrogen bonds and hydrophobic interactions (Fig. 1B). The 7-deaza-adenine moiety of STM957 forms two hydrogen bonds with Tyr368 and one with Ala353, while the ethynyl linker bound to C7 forms hydrophobic interactions with Phe367. The central ribose part of the ligand forms two hydrogen bonds with Asp352 via its hydroxyl moieties. The *N*-ethyl substituent of sulfonamide group forms a hydrophobic interaction to Pro335, while the SO_2_ group is surface-exposed. The cyano substituent of the benzenesulfonamide part is hydrogen-bound to Asn388 and the methoxy group forms a hydrophobic interaction with Phe506.

**Fig. 2:**
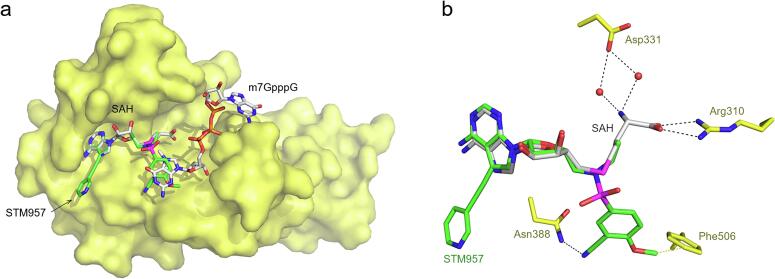
Comparison of STM957 and SAH binding to SARS-CoV-2 nsp14 a, Overall view of the superposition of STM957, SAH, and m7GpppG bound to nsp14 depicted as in Fig. 1A. SAH and m7GpppG were placed into the nsp14/STM957 structure using previously reported structures of the nsp14/SAH, pdb entry 7TW8 (Kottur et al., 2022), and nsp14/m7GpppG complex, pdb entry 7QIF (Imprachim et al., 2023), respectively. b, Detailed view of the superposition of STM957 and SAH bound to nsp14. Ligands and selected nsp14 amino acid residues are depicted as in Fig. 1B except for carbon atoms of SAH, which are colored in grey. Water molecules are shown as red spheres. (For interpretation of the references to colour in this figure legend, the reader is referred to the web version of this article.)

A high resolution of nsp14 bound to SAH is already available (Kottur et al., 2022). We compared the conformation of the SAH and STM957 (Fig. 2A). The bases are at virtually the same place and therefore the 7-deaza substituent was located at an expected position because it is linked to the base by a triple bond that does not allow for much conformational freedom. However, the benzenesulfonamide moiety occupies and a different position than the methionine moiety. While the methionine moiety forms hydrogen bonds with Arg310 and water bridges with Asp331 the benzenesulfonamide moiety forms a hydrogen bond with Asn338 and a hydrophobic interaction with Phe506. These findings are in excellent agreement with our previous docking studies (SI Fig. 1) (Stefek et al., 2023) conducted using two distinct docking software programs, Gold and Autodock Vina. Both approaches indicated that the positioning of the aromatic moiety linked to the sulfonamide bridge exhibits similar energy levels. Furthermore, these studies suggested that this part of the molecule binds at a site that is, during the enzymatic reaction, occupied by the guanine moiety (Fig. 2) of the viral RNA precap structure (Imprachim et al., 2023).

**Fig. 3:**
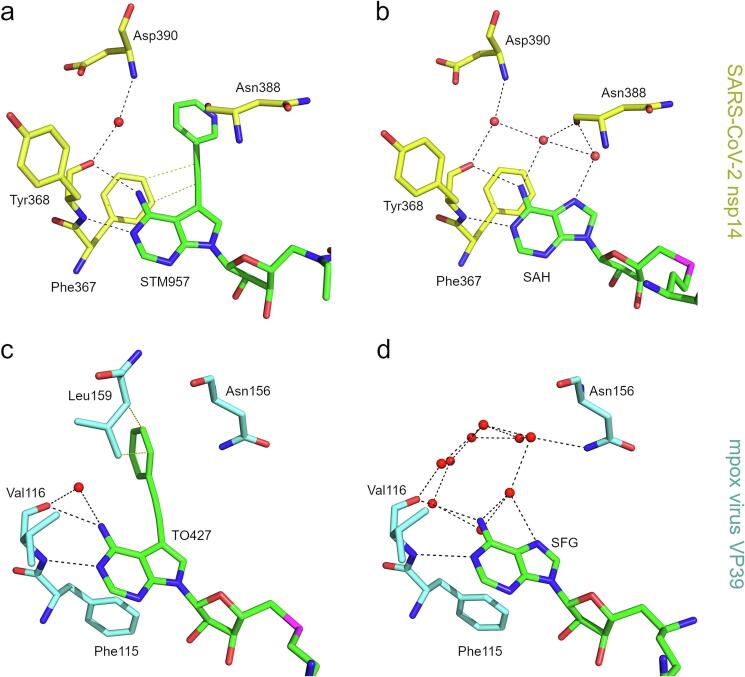
Displacement of water molecules with 7-deaza modified SAH analogs a, b, Detailed view of the water molecules coordinated next to the adenine bases of STM957 (a) and SAH (b), pdb entry 7TW8 (Kottur et al., 2022), bound to SARS-CoV-2 nsp14. c, d, Detailed view of the water molecules coordinated next to the adenine bases of TO427 (c), pdb entry 8CEQ (Silhan et al., 2023), and sinefungin (d), pdb entry 8B07 (Silhan et al., 2023) bound to mpox virus MTase VP39.

Previous structural analysis of nsp14 bound to SAH (Kottur et al., 2022) also revealed a small network of water molecules that mediate the interaction of nsp14 with the adenine base (Fig. 3). Interestingly, these water molecules are removed by the large 7-deaza substituent (Fig. 3). We have previously observed similar displacement of water molecules with SAH-based compounds bearing a large aromatic substituent at this position that were designed as inhibitor of the monkeypox MTase VP39 (Silhan et al., 2023, Zgarbova et al., 2023). Therefore, displacement of water molecules from the vicinity of the adenine base appears as a common mechanism for 7-deaza modified SAH analogs (Fig. 3) and surprisingly leads to sub-micromolar inhibitors of viral MTases from evolutionary distinct viral families such as the Coronaviridae and Poxviridae.

## CRediT authorship contribution statement

**Eva Zilecka:** Investigation. **Martin Klima:** Writing – original draft, Formal analysis, Conceptualization. **Milan Stefek:** Writing – original draft, Supervision, Resources, Data curation. **Milan Dejmek:** Data curation, Resources, Supervision, Writing – original draft. **Radim Nencka:** Resources, Funding acquisition, Conceptualization. **Evzen Boura:** Writing – original draft, Funding acquisition, Conceptualization.

## Declaration of competing interest

The authors declare that they have no known competing financial interests or personal relationships that could have appeared to influence the work reported in this paper.

## Data Availability

Data will be made available on request. The structure was also deposited in the PDB database under the accession code 9FEH.
