# Supplementary material for: Structure of SARS-CoV-2 MTase nsp14 with the inhibitor STM957 reveals inhibition mechanism that is shared with a poxviral MTase VP39
Source: J Struct Biol X. 2024 Jul 29;10:100109. doi: 10.1016/j.yjsbx.2024.100109 (PMC11345338; doi:10.1016/j.yjsbx.2024.100109)
Supplement: Supplementary Data 1 [file mmc1.pdf]

Structure of SARS-CoV-2 MTase nsp14 with the inhibitor STM957 reveals inhibition mechanism that is shared with a poxviral MTase VP39

Eva Zilecka[1], Martin Klima[1], Milan Stefek[1], Milan Dejmek[1], Radim Nencka[1], Evzen Boura[1,*]

[1]Institute of Organic Chemistry and Biochemistry, Academy of Sciences of the Czech Republic, v.v.i, Flemingovo nám. 2, 166 10 Prague 6, Czech Republic.

[*]correspondence to boura@uochb.cas.cz

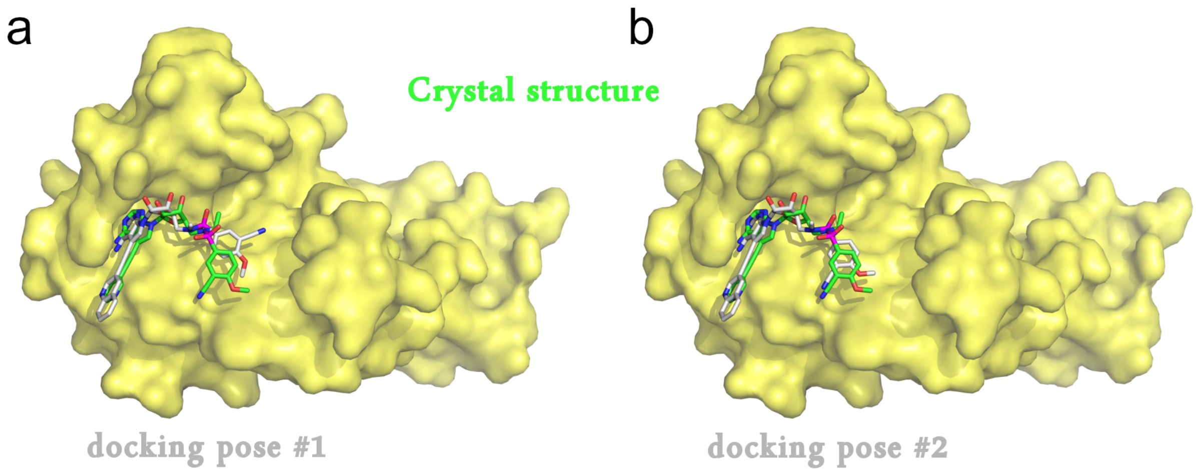

Supplementary Figure 1: Comparison of previous docking experiments (Stefek et al., 2023) and the actual crystal structure. a) The carbons of the ligand in docking pose #1 and b) the ligand in docking pose #2 are in grey. The carbons of the ligand from the crystal structure are in green. The position of the benzenesulfonamide part in the docking pose #2 is almost identical to the crystal structure.

Stefek, M., Chalupska, D., Chalupsky, K., Zgarbova, M., Dvorakova, A., Krafcikova, P., Li, A.S.M., Sala, M., Dejmek, M., Otava, T., Chaloupecka, E., Kozak, J., Kozic, J., Vedadi, M., Weber, J., Mertlikova-Kaiserova, H., Nencka, R., 2023. Rational Design of Highly Potent SARS-CoV-2 nsp14 Methyltransferase Inhibitors. ACS Omega 8, 27410-27418.
